# Pre-gastrula expression of zebrafish extraembryonic genes

**DOI:** 10.1186/1471-213X-10-42

**Published:** 2010-04-27

**Authors:** Sung-Kook Hong, Carly S Levin, Jamie L Brown, Haiyan Wan, Brad T Sherman, Da Wei Huang, Richard A Lempicki, Benjamin Feldman

**Affiliations:** 1 Medical Genetics Branch, National Human Genome Research Institute, National Institutes of Health, Bethesda, MD 20892, USA; 2 Laboratory of Immunopathogenesis and Bioinformatics, Clinical Services Program, SAIC-Frederick, Inc., National Cancer Institute at Frederick, Frederick, MD 21702, USA

## Abstract

**Background:**

Many species form extraembryonic tissues during embryogenesis, such as the placenta of humans and other viviparous mammals. Extraembryonic tissues have various roles in protecting, nourishing and patterning embryos. Prior to gastrulation in zebrafish, the yolk syncytial layer - an extraembryonic nuclear syncytium - produces signals that induce mesoderm and endoderm formation. Mesoderm and endoderm precursor cells are situated in the embryonic margin, an external ring of cells along the embryo-yolk interface. The yolk syncytial layer initially forms below the margin, in a domain called the external yolk syncytial layer (E-YSL).

**Results:**

We hypothesize that key components of the yolk syncytial layer's mesoderm and endoderm inducing activity are expressed as mRNAs in the E-YSL. To identify genes expressed in the E-YSL, we used microarrays to compare the transcription profiles of intact pre-gastrula embryos with pre-gastrula embryonic cells that we had separated from the yolk and yolk syncytial layer. This identified a cohort of genes with enriched expression in intact embryos. Here we describe our whole mount *in situ *hybridization analysis of sixty-eight of them. This includes ten genes with E-YSL expression (*camsap1l1*, *gata3*, *znf503*, *hnf1ba*, *slc26a1*, *slc40a1*, *gata6*, *gpr137bb*, *otop1 *and *cebpa*), four genes with expression in the enveloping layer (EVL), a superficial epithelium that protects the embryo (*zgc:136817*, *zgc:152778*, *slc14a2 *and *elovl6l*), three EVL genes whose expression is transiently confined to the animal pole (*elovl6l*, *zgc:136359 *and *clica*), and six genes with transient maternal expression (*mtf1*, *wu:fj59f04*, *mospd2*, *rftn2*, *arrdc1a *and *pho*). We also assessed the requirement of Nodal signaling for the expression of selected genes in the E-YSL, EVL and margin. Margin expression was Nodal dependent for all genes we tested, including the concentrated margin expression of an EVL gene: *zgc:110712*. All other instances of EVL and E-YSL expression that we tested were Nodal independent.

**Conclusion:**

We have devised an effective strategy for enriching and identifying genes expressed in the E-YSL of pre-gastrula embryos. To our surprise, maternal genes and genes expressed in the EVL were also enriched by this strategy. A number of these genes are promising candidates for future functional studies on early embryonic patterning.

## Background

Extraembryonic tissues have transient functions to protect, nourish and pattern embryos during embryogenesis, but their cellular descendants are not incorporated into the adult body. Extraembryonic tissues are distinct from maternal tissues in that they are either part of the primary oocyte, for instance the yolk, or produced by embryonic cells. Examples of human extraembryonic tissues are the placenta and its trophoblast cell progenitors, as well as the yolk sac and its hypoblast cell progenitors [1]. In teleost fish, the yolk, the yolk syncytial layer (YSL) and the enveloping layer (EVL) have long been considered to be extraembryonic [2,3]. This definition may need to be revised for the zebrafish EVL, as well as for the mouse hypoblast, because it was recently shown that some descendants of each of these tissues are incorporated into the adult body [4,5].

Zebrafish EVL cells first form at two hours post fertilization (hpf) [6]. This and the formation of primordial germ cells are the earliest differentiation steps in zebrafish embryogenesis [7]. EVL cells form a superficial epithelium that surrounds and protects embryonic cells, and the EVL is ultimately anchored to the YSL [2,8].

The YSL forms between the yolk and overlying embryonic cells at around 2.5 hpf, during the mid-blastula transition, when activation of the zygotic genome occurs [9]. The YSL forms via the collapse of a subset of embryonic margin cells, which are the outermost embryonic cells adjacent to the yolk. This produces an aqueous extraembryonic layer between the lipid-rich yolk and the embryo. This layer is comprised of multiple nuclei, other cellular organelles and a dense mesh of cytoskeletal proteins. YSL nuclei are initially restricted to the exterior of the yolk, in a region called the external YSL (E-YSL). The E-YSL nuclei undergo several rounds of synchronous division. At the onset of epiboly, which is the first concerted movement of embryonic cells, a number of YSL nuclei spread inwards to occupy the interior yolk-embryo interface, a region called the inner YSL (I-YSL) [10].

YSL nuclei are transcriptionally active. Evidence for this is found in genes like *mxtx1 *that are expressed exclusively in the YSL [11]. The YSL layer is also able to translate mRNA into protein. For instance, injection of an mRNA encoding the Nodal-related 1 (Ndr1) ligand into the YSL leads to the activation of downstream targets in marginal embryonic cells[12].

The YSL and EVL have several functions in development. These include the YSL's processing and delivery of nutritive lipids from the yolk, the EVL's role as a permeability barrier, and the action of both tissues in driving epiboly [8,13-15]. The YSL of blastula-stage embryos also produces signals that are necessary and sufficient for inducing ventrolateral mesoderm and endoderm (mesendoderm) in the embryonic margin [16,17]. YSL sufficiency is inferred from the ability of transplanted yolk cells to induce mesoderm gene expression, and YSL necessity is inferred from the loss of mesendoderm gene expression in embryos whose YSL is globally depleted of mRNAs. Expression of the Nodal-related ligands *ndr1 *and *ndr2 *in the YSL account for some - but not all - of this activity [18,19]. Because the E-YSL is located directly below the embryonic margin, we hypothesize that genes with key roles in the induction of ventrolateral mesoderm and endoderm are expressed in this domain during early blastula stages.

We sought to identify genes with E-YSL expression. To generate a list of genes with an enhanced probability of E-YSL expression, we used microarrays to compare gene expression levels in blastula-stage whole embryos relative to FACS-purified embryonic cells that had been stripped free of the yolk and YSL. We examined the expression of sixty-eight genes enriched in whole embryos, using whole mount *in situ *hybridization (WISH). This revealed a substantial number of genes with E-YSL expression. To our surprise, EVL and maternally expressed genes were also enriched by this procedure.

## Results

### Enrichment of YSL, EVL and maternal genes by comparative microarray

**Figure 1:**
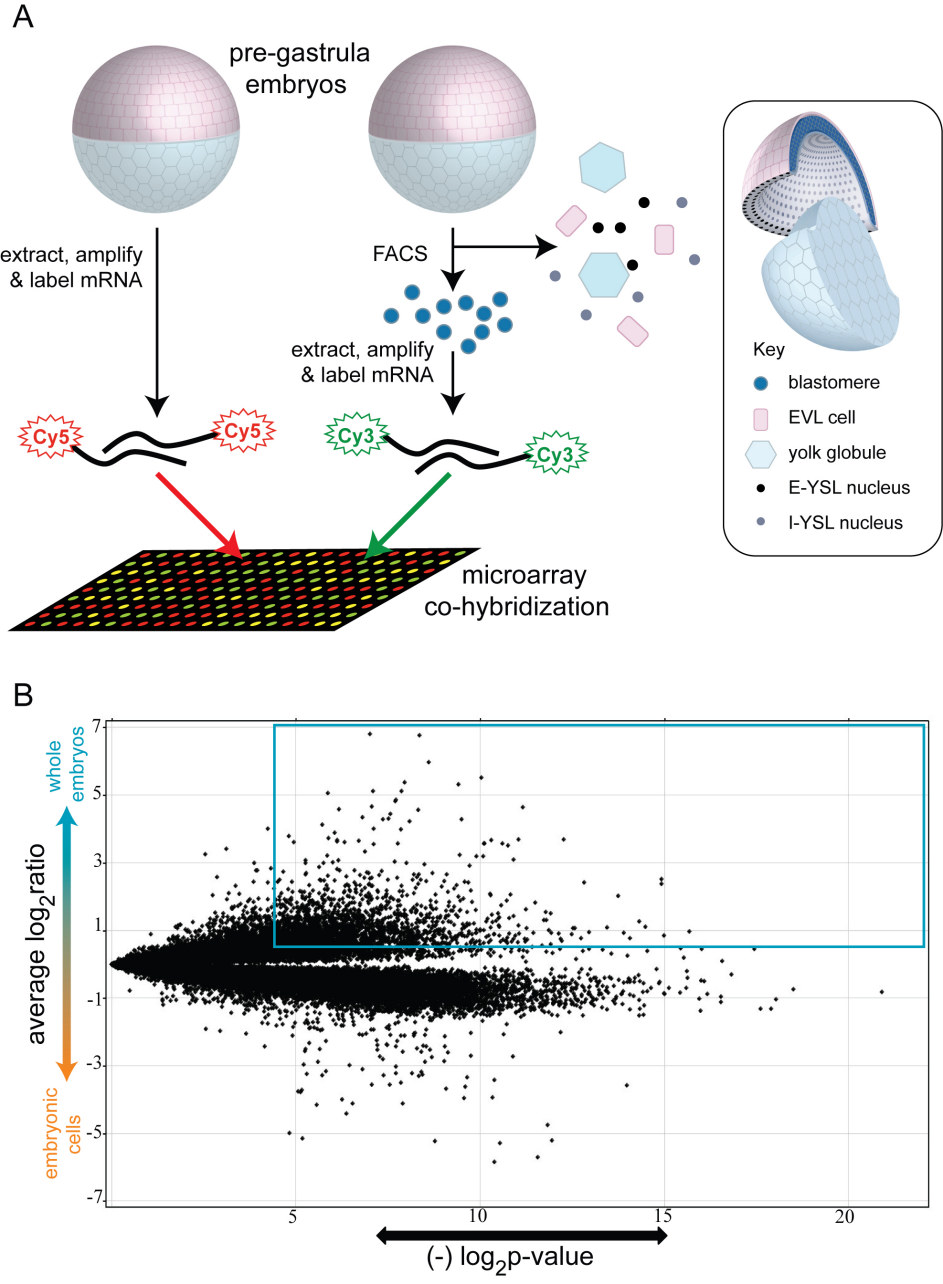
**Strategy for enrichment of transcripts expressed in the yolk syncytial layer**. (1A) schematic of strategy. Embryos were injected with purified Kaede protein after fertilization, labelling all cells green, and incubated until the blastula stage (5 hpf - "pre-gastrula embryos" in schematic), then either directly harvested for mRNA extraction and probe generation, or FACS sorted followed by mRNA extraction and probe generation. Differentially labeled probes from these two procedures were co-hybridized onto microarrays. The exclusion of yolk globules was observed in microscopic examinations of post-FACS samples (data not shown), and the exclusion of YSL nuclei and EVL is inferred from subsequent analyses described in this paper. (1B) volcano plot representation of microarray hybridization. A substantial number of genes with significantly elevated transcript levels are apparent, both in the whole embryo (537 oligos outlined by the blue box which correspond to 359 unique genes) and embryonic cell cohorts (1697 oligos which correspond to 1210 unique genes, using analogous criteria, see Methods).

We hypothesize that the zebrafish E-YSL is a likely source of key signals for inducing ventrolateral mesendoderm. This hypothesis is based on experimental embryology studies and on the E-YSL's proximity to prospective ventrolateral mesendoderm [9,16,17,20,21]. To identify genes expressed in the E-YSL, we prepared cRNA probes from intact blastula-stage embryos and co-hybridized these with differentially labelled cRNA probes from embryonic cells that had been disaggregated and FACS purified, rendering them free of the yolk (Figure 1A). Embryonic cells in both groups had been labeled green via embryonic injection of purified Kaede protein, facilitating FACS of the disaggregated group. This microarray comparison yielded 359 unique genes with significantly higher expression levels in whole embryos (Figure 1B). We refer to this as the *whole vs. green *cohort (Figure 1B; NCBI GEO, accession #GSE8654).

**Figure 2:**
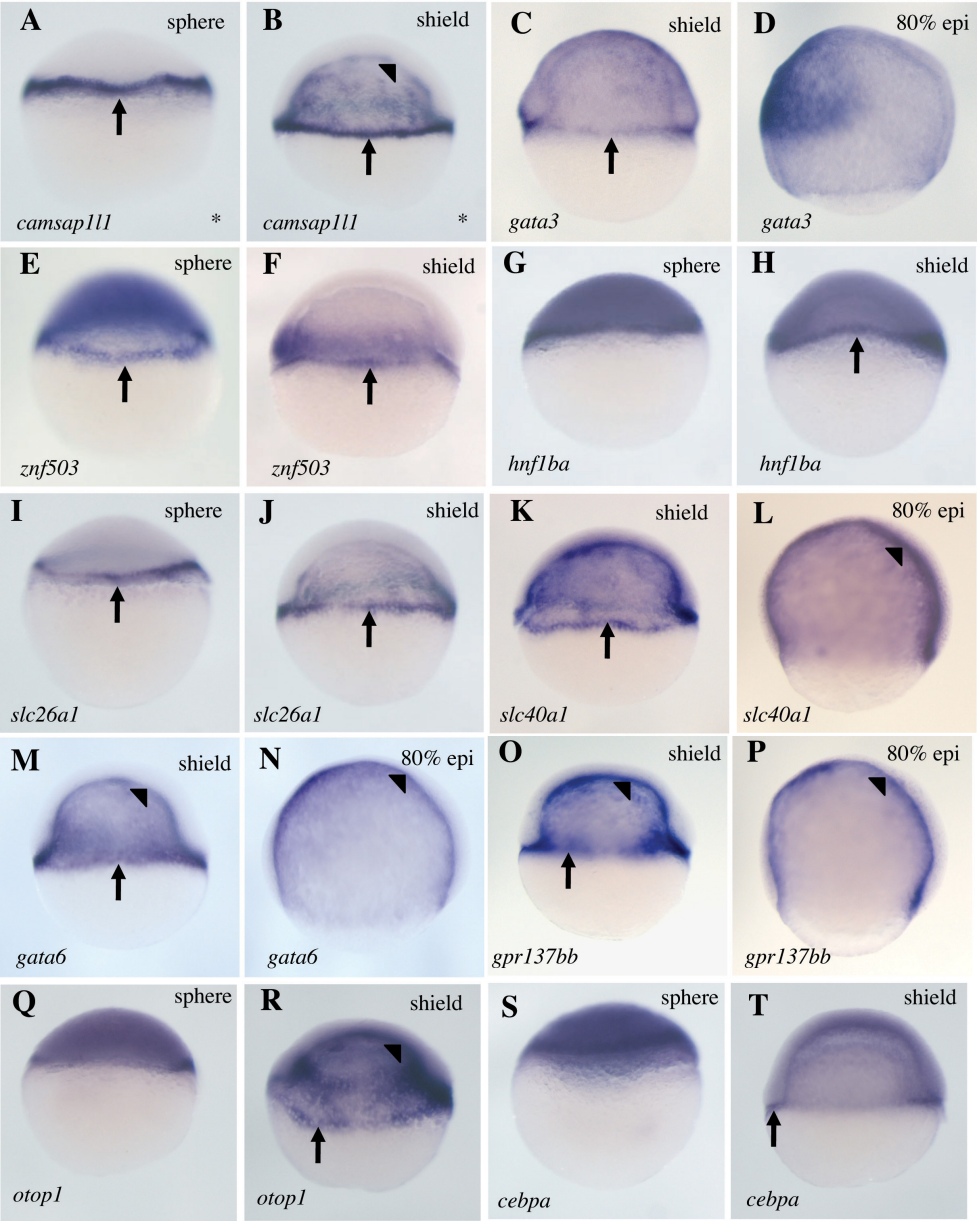
**Expression of genes in the E-YSL**. Whole mount *in situ *hybridizations showing E-YSL expression of mRNAs encoding the transcription factors *gata3 *[48], *znf503 *[49-51], *hnf1ba *[52], *gata6 *[30] and *cebpa *[30,53], one cytoskeletal protein that is potentially associated with the inner plasma membrane (*camsap1l1 *[54]), two solute carriers (*slc26a1 *[30] and *slc40a1 *[30]), one G-protein coupled receptor (*gpr137bb *[55]) and one integral membrane protein (*otop1 *[31,56,57]). Lateral views are presented throughout, with the dorsal side (where apparent) to the right for shield and 80% epiboly stages. Developmental stages and genes probed are indicated. Arrows point to E-YSL expression and arrowheads point to I-YSL expression. Asterisks on the bottom right corner indicate genes for which no *in situ *expression data has previously been reported.

**Figure 3:**
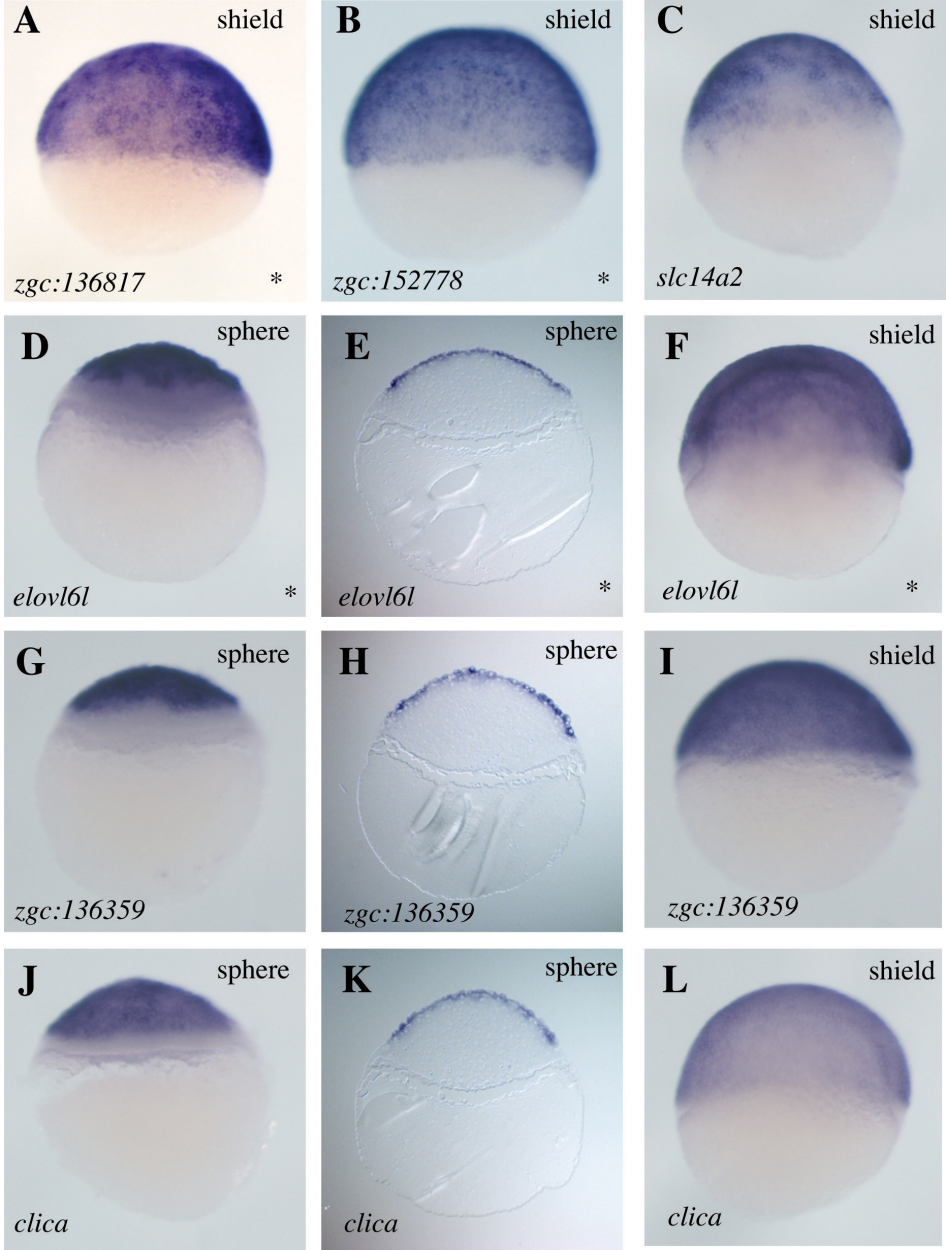
**Expression of genes in the EVL**. Whole mount *in situ *hybridizations for mRNAs expressed in the EVL encoding one RhoGEF (*zgc:136817 *[58]), one protein with a LIM zinc binding domain (*zgc:152778 *[58]), one solute carrier (*slc14a2 *[59]), one protein related to a family of long chain fatty acid-elongating enzymes (*elovl6l *[58]), one protein with a DEP domain (*zgc:136359 *[31]), indicating it might be involved in G-protein coupled receptor recognition, and one intracellular chloride channel protein (*clica *[31]). Expression of three of these genes was excluded from the margin at the sphere stage of development (D, G and J), also seen in histological sections (E, H and K). By shield stage, expression is apparent throughout the EVL (F, I and L). Lateral views are shown throughout and developmental stages and genes probed are indicated. Asterisks on the bottom right corner indicate genes for which no *in situ *expression data has previously been reported.

**Figure 4:**
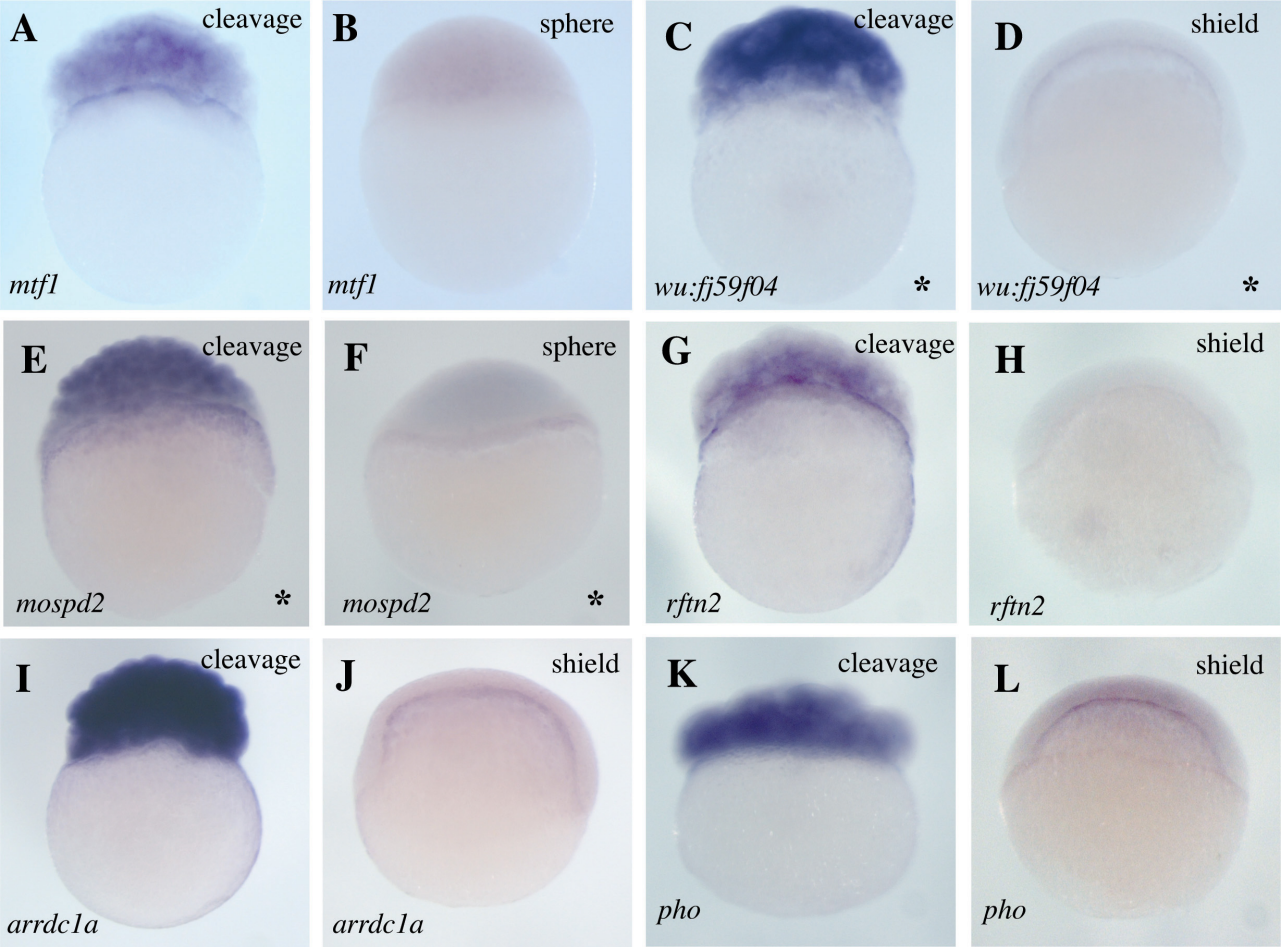
**Expression of transient maternal genes**. Whole mount *in situ *hybridizations showing what we presume to be maternal expression for mRNAs encoding one transcription factor (*mtf1 *[60]), two proteins with no recognizable motifs (*wu:fj59f04 *and *pho *[61]), one protein with similarity to the human *MOTILE SPERM DOMAIN CONTAINING 2 *gene (*mospd2*), one protein belonging to a lipid raft-linking family (*rftn2 *[31]) and one protein with an arrestin domain (*arrdc1a *[30]). Lateral views are shown and developmental stages and genes probed are indicated. Asterisks on the bottom right corner indicate genes for which no *in situ *expression data has previously been reported.

As evidence that our strategy had worked, three genes previously reported to have E-YSL expression were significantly enriched by this procedure: *mxtx1*, *mxtx2 *and *hnf4a *(Additional File 1)[11,22]. To identify more such genes, we cloned and performed WISH on sixty-eight of the most enriched genes from the *whole vs. green *cohort. These included twenty-three genes whose expression has not previously been described. We assessed the expression of these sixty-eight genes at four developmental stages: cleavage (1-1.5 hpf), sphere (4 hpf), shield (6 hpf) and 80% epiboly (8 hpf). This revealed previously unreported expression for ten genes in the E-YSL (Figure 2). We also observed various other expression patterns (Additional Files 1 and 2), including a high incidence of genes expressed in the EVL (Figure 3) and genes expressed during cleavage stages, which we presume to be maternal (Figure 4).

### Expression of YSL, EVL and maternal genes

Figure 2 shows ten genes with E-YSL expression at sphere or shield stages. This is the first report of E-YSL expression for these genes, although developmental expression profiles for all but one - *camsap1l1 *- have previously been described. Half of these genes encode transcription factors. This is consistent with the preponderance of transcription factors among genes already known to be expressed in the blastula E-YSL (see Discussion). We noted I-YSL expression for *cammsap1l1, slc4a1, gata6, gpr137bb, otop1 *and *cebpa*, but not for *gata3*, *znf503*, *hnf1ba *or *slc26a1*. We also identified a number of genes with I-YSL but no E-YSL expression (Additional Files 1 and 2).

Figure 3 presents data for six genes whose EVL expression has not previously been described. We noted restriction of EVL expression to the animal pole of sphere stage embryos for three of these genes: *elovl6l, zgc:136359 *and *clica*. This restriction is temporal: each of these three genes is expressed throughout the EVL by shield stage. We saw no bias in the types of proteins encoded by these genes.

Forty-four of the sixty-eight genes we examined showed expression during cleavage stages (1-1.5 hpf) (Additional File 1). These mRNAs are presumed to be maternal in origin, because only a small fraction of zygotic genes are expressed prior to the midblastula transition at 2.5 hpf [23]. Six of these genes showed a maternal-only pattern of expression (Figure 4). No bias in molecular class was seen for these maternal-only genes.

### Regulation of an EVL gene, but not YSL genes, by Nodal signaling

**Figure 5:**
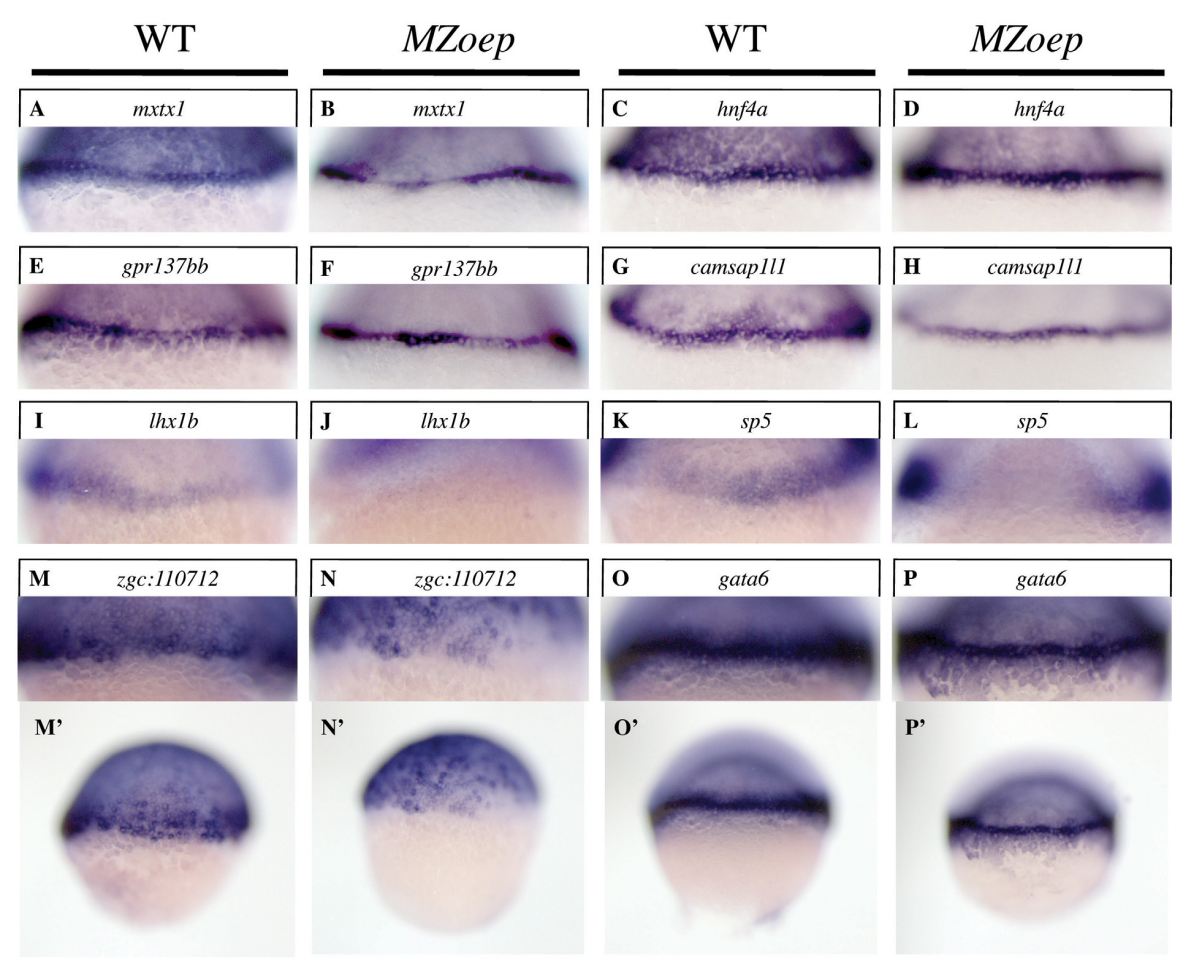
**Nodal-independent regulation of genes in the YSL**. Whole mount *in situ *hybridizations on WT and *MZoep *embryos at the early-gastrula (6 hpf) stage. Lateral magnifications of the margin and YSL are shown throughout, except for lateral whole-embryo views in M', N', O' and P'. No significant difference was seen in the YSL expression levels of *mxtx1 *in WT or *MZoep *embryos, as was previously reported (A-B). Similarly, no significant difference was seen between WT and *MZoep *embryos for the YSL expression of *hnf4a *(C-D), *gpr137bb *(E-F), or *camsap1l1 *(G-H). The expression of *lhx1b *(I-J) and *sp5 *(K-L) was substantially reduced in *MZoep *mutants. Similarly, concentrated expression of *zgc:110712 *near the margin was absent in *MZoep *embryos (M, M', N, N'). The *gata6 *gene is expressed both in the YSL and margin (O and O') and the margin expression domain is eliminated in *MZoep *mutants, whereas the YSL expression domain persists (P and P').

We assessed the Nodal regulation of several YSL, EVL and margin genes identified in this and a previous screen [24]. As expected from previous reports, expression of the two margin genes we tested was downregulated in the margin of *MZoep *mutant embryos, which carry null mutations in an EGF-CFC-type receptor that is essential for Nodal signaling (Figure 5I-L) [25,26]. In the case of *sp5*, a zinc-finger transcription factor gene, this loss was limited to the dorsal domain (Figure 5K-L). Interestingly, the EVL gene *zgc:110712*, encoding a keratin, also lost its concentrated expression in the margin of *MZoep *embryos (Figure 5M, M', N, N') [24].

Consistent with reports for other YSL genes (*i.e., mxtx1*, *mxtx2 *and *sox32*), the YSL expression of *hnf4a*, *gpr137bb *and *camsap1l1 *was unchanged in *MZoep *embryos (Figure 5C-H) [11,27-29]. The *gata6 *gene [30] also showed persistent expression in the YSL, however there was a visible narrowing and sharpening of its expression domain (Figure 5O, O', P, P'), indicating that like *sox32*, *gata6 *is expressed in both the margin and YSL, and is lost exclusively from the margin of Nodal-deficient embryos.

We also examined Nodal regulation of *elovl6l, zgc:136359 *and *clica*, whose expression is excluded from the EVL margin at sphere stage (Figure 3). We asked whether exclusion of these genes from the margin might be due to early repression by nascent Nodal signals. However we saw no change in the expression of *elovl6l, zgc:136359 *or *clica *in *MZoep *embryos, indicating that these genes are not repressed by Nodal signals (data not shown).

## Discussion

### Identification of genes with E-YSL expression domains

We have implemented a strategy that enriches for genes expressed in the YSL, including the E-YSL. Our documentation of previously unrecognized E-YSL expression for ten genes increases the number of candidates for involvement in early YSL functions. With respect to mesendoderm induction, those E-YSL genes (*i.e*., *camsap1l1*, *znf503 *and *slc26a1*) that are expressed at the sphere stage (4 hpf) or earlier are of greater interest, because this is the critical phase for induction [16]. Our literature and database searches found documentation of E-YSL expression for five additional genes at 4 hpf or earlier: *apoeb *[30], *fgf17 *[31], *mxtx1 *and *mxtx2 *[11] and *sox32 *[29]. There is also antisense morpholino-based loss of function evidence that expression of *ndr1 *and *ndr2 *in the early E-YSL accounts for some of the YSL's endoderm-inducing activity [18].

Our microarray study was performed on 5 hpf embryos, and therefore should also have enriched for E-YSL genes expressed at this stage. A literature and database search extending to 5 hpf includes the aforementioned *apoeb*, *fgf17*, *mxtx1*, *mxtx2 *and *sox32 *and adds *gata5 *[32], *fbp1b *[33], *bcl2l10 *[34], *cdh2 *[35], *hhex *[36], *ndr2 *[18] and *gbx1 *[37] to the list of genes with E-YSL expression. We re-identified *mxtx1 *and *mxtx2*, but none of the others (Additional Files 1 and 2 and data not shown). Detection of *fgf17 *and *fbp1b *was not possible for the trivial reason that they are not represented on our microarray. Our failure to detect *sox32*, *gata5*, *ndr2*, *gbx1 *and *apoeb *may be due to the prominent co-expression of these genes by embryonic cells, as reported in 4 to 6 hpf embryos, which could have prevented their differential detection in the *whole vs. green *comparison (Figure 1A). The remaining three E-YSL genes, *bcl2l10*, *cdh2 *and *hhex*, were enriched in our study, but excluded by our statistical cutoff, indicating variability in signal intensity between repeated microarray hybridizations (see Materials and Methods).

A substantial fraction of early E-YSL genes encode transcription factors, including those reported in this study. The blastoderm of *Drosophila *embryos is another early syncytium that is rich in transcription factors. These *Drosophila *transcription factors orchestrate the conversion of gene expression domains from broad gradients to discrete stripes, through gene regulatory networks and trans-nuclear regulation [38,39]. It will be interesting to learn whether analogous patterning mechanisms occur in vertebrate nuclear syncytia, such as the YSL of teleosts or the syncytiotrophoblast of mammalian placentae.

### EVL and maternal genes

Our strategy unexpectedly yielded EVL genes and maternal genes. We confirmed that the enrichment for EVL genes is statistically significant through the following analysis. We mined a database (the Zebrafish Information Network) that has text terms describing the developmental expression of more than 10,000 zebrafish genes [40]. The term "EVL" was associated with 10.2% (49) of the 478 top enriched genes from the *whole vs. green *cohort, but only with 2.3% (136) of 5,901 genes tested from across the entire microarray. We determined that this is a significant enrichment (P = 7.1E-20), using a Z test. We did not find a way to quantify the significance of the apparent maternal gene enrichment. However, our subjective comparison to other expression screens convinces us that 44 maternal genes out of 68 examined is substantially higher than random [22,30,31,33].

We are not certain why these EVL and maternal gene enrichments occurred. However we hypothesize that the EVL enrichment arose from an exclusion of EVL cells during the FACS procedure. This possibility is schematized in Figure 1A. Regarding maternal genes, perhaps disaggregation and FACS caused a downregulation of maternal mRNAs that did not occur in whole embryos. Alternatively, perhaps maternal mRNA is diffusely retained in the yolk without being degraded, and this maternal mRNA pool was lost in the FACS steps, causing higher levels in whole embryos.

The EVL and maternal genes we have described should be of interest for future studies. The animal pole restriction of EVL that we observed for *elovl6l, zgc:136359 *and *clica*, and their subsequent expression throughout the EVL at shield stage, suggests the existence of an animal-margin progression of EVL differentiation. This gradient is opposite to and independent of the Nodal-driven margin-animal progression of germ layer differentiation [41]. We did not detect any cleavage-stage expression for *zgc:136359 *(Additional File 2C), indicating that this gene's initiation of expression may be concomitant with EVL formation. By contrast, both *elovl6l *and *clica *display ubiquitous expression at the cleavage stage (Additional File 2). This suggests that a rapid decay or redistribution of maternal transcripts for these two genes occurs between the cleavage and sphere stage.

The maternal control of vertebrate embryogenesis is a topic with many open questions, owing to a lack of robust strategies for identifying essential maternal factors. Nonetheless, a sufficient number of essential maternal genes have been described to establish that maternal factors have a critical role in vertebrate embryonic development, including fish and mammals [42-44]. One step towards understanding the maternal control of zebrafish embryogenesis will be defining its maternal transcriptome. Here we document the maternal expression of thirty-five genes that, to the best of our knowledge, have not previously been reported (Additional Files 1 and 2). We highlight an intriguing pattern of maternal-only expression for six genes that may have roles at the onset of embryogenesis.

### Nodal regulation of extraembryonic tissues

We found expression of *zgc:110712 *in the EVL margin to be Nodal dependent. The contribution of specialized EVL cells to Kupffer's vesicle has also been shown to be Nodal dependent [5]. Finally, a regulatory element from the *ndr1 *promoter was shown to drive GFP expression specifically in the EVL [18]. Thus, Nodal signals clearly regulate aspects of EVL gene expression and behavior.

By contrast, the YSL appears to function independently of Nodal signaling. The data we present here increases the number of documented genes with Nodal-independent YSL expression from three to seven, with no instances of Nodal-dependent YSL expression reported. This is consistent with a recent study showing that a reporter of Smad2/Smad4 dimerization, which is an essential step for Nodal signaling, is not activated in the YSL [45]. This lack of conventional Nodal signaling in the YSL indicates that distinct pathways must be employed to drive expression of genes in the YSL. This restriction should apply to the expression of *ndr1 *and *ndr2 *themselves: although Ndr1 and Ndr2 can induce their own transcription in most of the embryo, this is likely not an option in the YSL [46]. Given that Ndr1 and Ndr2 in the YSL account for some of the YSL's mesendoderm-inducing activity, elucidating how they become activated in the YSL is a critical goal.

## Conclusions

Our quest for genes expressed in the YSL of early-stage embryos has uncovered E-YSL expression, EVL expression and transient maternal expression for a score of genes. Several of these genes are excellent candidates for future studies on the early embryonic patterning of zebrafish. Our understanding of these early patterning events must take into account evidence from this study and others that gene expression in the YSL is refractory to Nodal signaling. The signaling networks present in the YSL and how they pattern the overlying mesoderm and endoderm remain unclear. Utilization of anatomical enrichment techniques such as those presented in this study, in combination with the powerful genetics that zebrafish provide should prove fruitful in elucidating these processes.

## Methods

### Fish stocks, mutants and embryo production

WT AB zebrafish were used for all embryo production, except for *MZoep *embryos, which were derived from crosses of *oep*^tz257/tz257 ^males and females that had been rescued via injection of ~50 pg WT *oep *mRNA, as previously described [25]. For crosses, up to three males and three females were placed together in the morning and embryos were collected immediately after spawning. Incubations were at 28.5 degrees or room temperature, and staging was according to Kimmel [3].

### Generation of the Whole embryo gene cohort

Embryos were injected with 3.5 ng purified Kaede protein, then dechorionated and half were manually dissociated, chilled and subjected to FACS purification optimized to collect only the embryonic cells based on their morphological properties and fluorescence. These embryonic cells were lysed in TRIzol. The other half of the embryos that were left intact were subjected to parallel incubation temperatures and lysed in TRIzol at the same time. Amplified mRNA from the FACS-sorted cells and the whole embryos was differentially labeled and co-hybridized to NHGRI's custom-made zebrafish microarray. This experiment was performed in parallel to a previous study on margin-specific genes [24]. Experimental details on the microarray and statistical analyses are therefore the same as described, except that three rather than four biological replicates were performed [24](NCBI GEO, accession # GSE8654). Elimination of oligos showing multiple hypothesis-corrected (Benjamini-Hochberg method) P values greater than 0.05 or fold enrichment values below 1.5 yielded 537 oligos out of a total of 33,897 on the microarray. These 537 oligos mapped to 359 genes in a May 2007 analysis.

### Gene cloning, WISH and photography

We selected and cloned 68 genes of those with the highest enrichment scores into pGEMT-Easy TA vectors (Promega) after PCR amplification from cDNA. Cleavage, sphere, shield, and 80% epiboly stage embryos were fixed in 4% paraformaldehyde for WISH [47]. Stained embryos were cleared through a glycerol series and photographed.

## Authors' contributions

S-KH Designed experiments and performed all cloning, WISH and photography for WT embryos. CSL performed WISH and photography for the Nodal regulation experiments. JLB. performed the *whole vs. green *microarray study and helped write the manuscript. HW performed WISH for the Nodal regulation experiments. BTS, DWH and RAL performed the bioinformatic analysis of EVL gene incidence. BF designed experiments, guided research and wrote the manuscript. All authors have read and approved the final manuscript.

## Supplementary Material

Additional file 1 **Summary of whole-mount *in situ *hybridization screen and microarray statistics**. For each gene the following information is provided: the official gene symbol, a text summary of WISH expression at the four stages tested, the fold enrichment and Benjamini-Hochberg-corrected P values (P-corr) from our microarray experiments, the location of WISH images in the main text or Additional File 2 (AF2), and a citation for the earliest published WISH stage we could find. Genes are grouped into six categories: genes with E-YSL expression, genes with EVL expression, genes with transient maternal expression, genes with other spatiotemporally-restricted expression patterns, genes with ubiquitous expression and genes with no detected expression. The following abbreviations are used for the text descriptions of expression: ubqt (ubiquitous), vnt (ventral), drs (dorsal), anm (animal), vgt (vegetal), mrgn (margin), prx (paraxial), sctr (sector), non-hom (non-homogenous). When terms are placed in parentheses in the table, this reflects our judgment that the indicated stain may be a background artefact. Click here for file

Additional file 2 **Whole-mount in situ hybridization images for 56 genes**. Two hundred and forty images representing four stages with some multiple views for each of fifty-six genes are presented on 11 pages (A through K). The location of data for particular genes is indicated in Additional File 1. No data is shown for ten genes for which no expression was detected and two genes that showed ubiquitous expression. Click here for file
