# Supplementary material for: Pre-gastrula expression of zebrafish extraembryonic genes
Source: BMC Dev Biol. 2010 Apr 27;10:42. doi: 10.1186/1471-213X-10-42 (PMC2873407; doi:10.1186/1471-213X-10-42)
Supplement: Additional file 1 — Summary of whole-mount in situ hybridization screen and microarray statistics. For each gene the following information is provided: the official gene symbol, a text summary of WISH expression at the four stages tested, the fold enrichment and Benjamini-Hochberg-corrected P values (P-corr) from our microarray experiments, the location of WISH images in the main text or Additional File 2 (AF2), and a citation for the earliest published WISH stage we could find. Genes are grouped into six categories: genes with E-YSL expression, genes with EVL expression, genes with transient maternal expression, genes with other spatiotemporally-restricted expression patterns, genes with ubiquitous expression and genes with no detected expression. The following abbreviations are used for the text descriptions of expression: ubqt (ubiquitous), vnt (ventral), drs (dorsal), anm (animal), vgt (vegetal), mrgn (margin), prx (paraxial), sctr (sector), non-hom (non-homogenous). When terms are placed in parentheses in the table, this reflects our judgment that the indicated stain may be a background artefact. [file 1471-213X-10-42-S1.PDF]

Gene Symbol	mat. WISH	sphere WISH	shield WISH	80% WISH	Log_2 Enrich.	P-corr	Figs	Earliest reported WISH stage, Reference
**eYSL expression**								
camsap1l1	-	eYSL	eYSL, iYSL	iYSL	4.319	0.048	2, 5, AF2A	No WISH report
cebpa	ubqt	ubqt	ubqt, eYSL, (iYSL)	ubqt	4.449	0.047	2, AF2A	80%, Thisse, B., Pflumio, S., Fürthauer, M., Loppin, B., Heyer, V., Degrave, A., Woehl, R., Lux, A., Steffan, T., Charbonnier, X.Q. and Thisse, C. (2001) Expression of the zebrafish genome during embryogenesis (NIH R01 RR15402). ZFIN Direct Data Submission.
gata3	-	ubqt	EVL, eYSL	vnt-anm-EVL	2.644	0.050	2, AF2E	80%, Grinblat, Y., Gamse, J., Patel, M., and Sive, H. (1998) Determination of the zebrafish forebrain: induction and patterning. Development 125: 4403-4416.
gata6	ubqt	ubqt	eYSL, iYSL, mrgn	iYSL	1.501	0.045	2,5, AF2H	80%, Thisse, B., Pflumio, S., Fürthauer, M., Loppin, B., Heyer, V., Degrave, A., Woehl, R., Lux, A., Steffan, T., Charbonnier, X.Q. and Thisse, C. (2001) Expression of the zebrafish genome during embryogenesis (NIH R01 RR15402). ZFIN Direct Data Submission.
gpr137bb	ubqt	ubqt	eYSL, iYSL	iYSL	1.129	0.043	2, 5, AF2D	80%, Thisse, B., Thisse, C. (2004) Fast Release Clones: A High Throughput Expression Analysis. ZFIN Direct Data Submission.
hnf1ba	ubqt	ubqt, eYSL	ubqt, eYSL	ubqt, vgt-prx	5.326	0.040	2, AF2A	80%, Lecaudey, V., Anselme, I., Rosa, F., and Schneider-Maunoury, S. (2004) The zebrafish Iroquois gene iro7 positions the r4/r5 boundary and controls neurogenesis in the rostral hindbrain. Development 131(13): 3121-3131.
hnf4a	-	-	eYSL, iYSL	iYSL	3.580	0.039	5, AF2B	30%, Kudoh, T., Tsang, M., Hukriede, N.A., Chen, X., Dedekian, M., Clarke, C.J., Kiang, A., Schultz, S., Epstein, J.A., Toyama, R., and Dawid, I.B. (2001) A gene expression screen in zebrafish embryogenesis. ZFIN Direct Data Submission.
mxtx1	-	eYSL	eYSL, iYSL	iYSL	5.980	0.043	5, AF2A	sphere, Hirata, T., Yamanaka, Y., Ryu, S.L., Shimizu, T., Yabe, T., Hibi, M., and Hirano, T. (2000) Novel mix-family homeobox genes in zebrafish and their differential regulation. Biochem. Biophys. Res. Commun. 271(3): 603-609.
mxtx2	(ubqt)	vgt	eYSL	-	3.703	0.034	AF2A	high , Hirata, T., Yamanaka, Y., Ryu, S.L., Shimizu, T., Yabe, T., Hibi, M., and Hirano, T. (2000) Novel mix-family homeobox genes in zebrafish and their differential regulation. Biochem. Biophys. Res. Commun. 271(3): 603-609.
otop1	ubqt	ubqt, eYSL	ubqt, eYSL, iYSL	ubqt, iYSL	2.759	0.049	2, AF2B	90%, Thisse, B., Thisse, C. (2004) Fast Release Clones: A High Throughput Expression Analysis. ZFIN Direct Data Submission.
slc26a1	-	eYSL	eYSL, iYSL	iYSL	3.102	0.038	2, AF2B	80%, Thisse, B., Pflumio, S., Fürthauer, M., Loppin, B., Heyer, V., Degrave, A., Woehl, R., Lux, A., Steffan, T., Charbonnier, X.Q. and Thisse, C. (2001) Expression of the zebrafish genome during embryogenesis (NIH R01 RR15402). ZFIN Direct Data Submission.
slc40a1	ubqt	ubqt	eYSL, iYSL	eYSL	1.370	0.046	2, AF2I	14-19 somites, Thisse, C., and Thisse, B. (2005) High Throughput Expression Analysis of ZF-Models Consortium Clones. ZFIN Direct Data Submission.
znf503	ubqt	ubqt, eYSL	mrgn	vgt-half	2.064	0.038	2, AF2E	30%, Hoyle, J., Tang, Y.P., Wiellette, E.L., Wardle, F.C., and Sive, H. (2004) nlz Gene family is required for hindbrain patterning in the zebrafish. Dev. Dyn. 229(4): 835-846.

Gene Symbol	mat. WISH	sphere WISH	shield WISH	80% WISH	Log_2 Enrich.	P-corr	Figs	Earliest reported WISH stage, Reference
**EVL expression**								
b3gnt5	-	-	EVL	EVL	1.208	0.045	AF2D	Dome, Cao, Y., Zhao, J., Wang, Y., and Meng, A. (2003) Expression of zebrafish Lc3 synthase gene in embryonic lens requires hedgehog signaling. Dev. Dyn. 228(3): 308-312.
clica	ubqt	anm-EVL	EVL	(ubqt)	1.961	0.049	3, AF2C	1 cell, Thisse, B., Thisse, C. (2004) Fast Release Clones: A High Throughput Expression Analysis. ZFIN Direct Data Submission.
elovl6l	EVL, ubqt	anm-EVL	EVL, drs-mrgn	(ubqt), (iYSL)	1.894	0.043	3, AF2C	No WISH report
osbpl7	EVL, ubqt	ubqt	EVL	EVL	1.687	0.045	AF2G	50%, Thisse, B., Thisse, C. (2004) Fast Release Clones: A High Throughput Expression Analysis. ZFIN Direct Data Submission.
si:ch211-157f15.1	-	EVL	EVL	EVL	1.710	0.050	AF2D	50%, Thisse, B., Pflumio, S., Fürthauer, M., Loppin, B., Heyer, V., Degrave, A., Woehl, R., Lux, A., Steffan, T., Charbonnier, X.Q. and Thisse, C. (2001) Expression of the zebrafish genome during embryogenesis (NIH R01 RR15402). ZFIN Direct Data Submission.
slc14a2	-	-	EVL	-	3.188	0.040	3, AF2B	prim-5, Braun, M.H., Steele, S.L., Ekker, M., and Perry, S.F. (2009) Nitrogen excretion in developing zebrafish (Danio rerio): A role for Rh proteins and urea transporters. Am. J. Physiol. Renal Physiol. 296(5): F994-F1005.
tfap2a	ubqt	anm-EVL	vnt-anm-EVL	vnt-anm-EVL	2.663	0.041	AF2B	50%, Thisse, B., Pflumio, S., Fürthauer, M., Loppin, B., Heyer, V., Degrave, A., Woehl, R., Lux, A., Steffan, T., Charbonnier, X.Q. and Thisse, C. (2001) Expression of the zebrafish genome during embryogenesis (NIH R01 RR15402). ZFIN Direct Data Submission.
zgc:136359	-	anm-EVL	EVL	vgt-prx-EVL	1.741	0.043	3, AF2C	90%, Thisse, B., Thisse, C. (2004) Fast Release Clones: A High Throughput Expression Analysis. ZFIN Direct Data Submission.
zgc:136817	ubqt	ubqt	EVL	EVL	1.327	0.045	3, AF2J	No WISH report
zgc:152778	-	-	EVL	EVL	2.960	0.044	3, AF2E	No WISH report

Gene Symbol	mat. WISH	sphere WISH	shield WISH	80% WISH	Log_2 Enrich.	P-corr	Figs	Earliest reported WISH stage, Reference
**Transient early expression (maternal only)**								
arrdc1a	ubqt	ubqt	-	-	1.115	0.031	4, AF2D	90%, Thisse, B., Pflumio, S., Fürthauer, M., Loppin, B., Heyer, V., Degrave, A., Woehl, R., Lux, A., Steffan, T., Charbonnier, X.Q. and Thisse, C. (2001) Expression of the zebrafish genome during embryogenesis (NIH R01 RR15402). ZFIN Direct Data Submission.
im:7147486	ubqt	ubqt	(ubqt)	(ubqt)	1.203	0.040	AF2D	1-cell, Thisse, B., Thisse, C. (2004) Fast Release Clones: A High Throughput Expression Analysis. ZFIN Direct Data Submission.
mospd2	ubqt	-	-	(iYSL)	1.723	0.044	4, AF2C	no WISH report
mtf1	ubqt	-	-	-	2.052	0.045	4, AF2F	1-cell, Chen, W.-Y., John, A.C.J., Lin, C.-H., and Chang, C.-Y. (2002) Molecular cloning and developmental expression of zinc finger transcription factor MTF-1 gene in zebrafish, Danio rerio. Biochem. Biophys. Res. Commun. 291(4): 798-805.
pho	ubqt	ubqt	(iYSL)	-	1.708	0.049	4, AF2F	long pec, Behra, M., Bradsher, J., Sougrat, R., Gallardo, V., Allende, M.L., and Burgess, S.M. (2009) Phoenix is required for mechanosensory hair cell regeneration in the zebrafish lateral line. PLoS Genet. 5(4): e1000455.
rftn2	ubqt	ubqt	-	-	2.338	0.040	4, AF2E	1-cell, Thisse, B., Thisse, C. (2004) Fast Release Clones: A High Throughput Expression Analysis. ZFIN Direct Data Submission.
wu:fj59f04	ubqt	ubqt	-	-	1.885	0.046	4, AF2F	no WISH report

Gene Symbol	mat. WISH	sphere WISH	shield WISH	80% WISH	Log_2 Enrich.	P-corr	Figs	Earliest reported WISH stage, Reference
**Other spatiotemporally-restricted expression patterns**								
aplnra	ubqt	ubqt, mrgn-sctr	ubqt, mrgn	ubqt, DEL	1.349	0.040	AF2J	oblong, Tucker, B., Hepperle, C., Kortschak, D., Rainbird, B., Wells, S., Oates, A.C., and Lardelli, M. (2007) Zebrafish Angiotensin II Receptor-like 1a (agtrl1a) is expressed in migrating hypoblast, vasculature, and in multiple embryonic epithelia. Gene Expr. Patterns 7(3): 258-265.
aqp3	ubqt	ubqt	ubqt, iYSL	ubqt, (iYSL)	1.598	0.040	AF2H	80%, Thisse, B., Thisse, C. (2004) Fast Release Clones: A High Throughput Expression Analysis. ZFIN Direct Data Submission.
cebpb	ubqt	ubqt	iYSL, mrgn	iYSL	1.443	0.046	AF2I	30%, Rauch, G.J., Lyons, D.A., Middendorf, I., Friedlander, B., Arana, N., Reyes, T., and Talbot, W.S. (2003) Submission and Curation of Gene Expression Data. ZFIN Direct Data Submission.
dazl	yolk, ubqt	yolk, ubqt, mrgn	ubqt	ubqt	1.472	0.040	AF2I	4-cell, Hashimoto, Y., Maegawa, S., Nagai, T., Yamaha, E., Suzuki, H., Yasuda, K., and Inoue, K. (2004) Localized maternal factors are required for zebrafish germ cell formation. Dev. Biol. 268(1): 152-161.
has2	(ubqt)	ubqt	mrgn	mrgn	1.179	0.042	AF2K	blastula, Bakkers, J., Kramer, C., Pothof, J., Quaedvlieg, N.E., Spaink, H.P., and Hammerschmidt, M. (2004) Has2 is required upstream of Rac1 to govern dorsal migration of lateral cells during zebrafish gastrulation. Development 131(3): 525-537.
hyou1	ubqt	ubqt	ubqt	(ubqt), iYSL	1.278	0.042	AF2J	80%, Thisse, B., Thisse, C. (2004) Fast Release Clones: A High Throughput Expression Analysis. ZFIN Direct Data Submission.
lmo4l	ubqt	ubqt, mrgn-sctr	(iYSL), drs-mrgn	vgt-drs	1.586	0.041	AF2H	80%, Thisse, B., Thisse, C. (2004) Fast Release Clones: A High Throughput Expression Analysis. ZFIN Direct Data Submission.
rnf103	ubqt	ubqt	(ubqt), drs-mrgn	-	1.699	0.041	AF2G	1 cell, Thisse, B., Thisse, C. (2004) Fast Release Clones: A High Throughput Expression Analysis. ZFIN Direct Data Submission.
scarb2	ubqt	ubqt	iYSL	iYSL	1.197	0.045	AF2K	1-4 somite, Thisse, C., and Thisse, B. (2005) High Throughput Expression Analysis of ZF-Models Consortium Clones. ZFIN Direct Data Submission.
smcr7	ubqt	ubqt	(ubqt), iYSL	(ubqt), iYSL	1.294	0.040	AF2J	1-cell, Thisse, B., Thisse, C. (2004) Fast Release Clones: A High Throughput Expression Analysis. ZFIN Direct Data Submission.
tnfrsf21	ubqt	ubqt	ubqt, iYSL	ubqt, (iYSL)	1.612	0.046	AF2G	75%, Eimon, P.M., Kratz, E., Varfolomeev, E., Hymowitz, S.G., Stern, H., Zha, J., and Ashkenazi, A. (2006) Delineation of the cell-extrinsic apoptosis pathway in the zebrafish. Cell Death Differ. 13(10): 1619-1630.
wu:fb59d01	ubqt	ubqt	(ubqt), iYSL	(ubqt), iYSL	1.289	0.049	AF2J	no WISH report
wu:fb94e12	(yolk) non-hom	ubqt	ubqt, iYSL	ubqt	1.656	0.048	AF2G	no WISH report
zgc:112184	ubqt	ubqt	ubqt	iYSL	1.180	0.046	AF2K	50%, Thisse, B., Thisse, C. (2004) Fast Release Clones: A High Throughput Expression Analysis. ZFIN Direct Data Submission.
zgc:136891	-	ubqt	ubqt, drs-mrgn	ubqt	3.574	0.040	AF2B	no WISH report
zgc:153073	(ubqt)	ubqt	ubqt, iYSL	ubqt, (iYSL)	2.017	0.032	AF2F	no WISH report

Gene Symbol	mat. WISH	sphere WISH	shield WISH	80% WISH	Log_2 Enrich.	P-corr	Figs	Earliest reported WISH stage, Reference
**Ubiquitous expression**								
cgnl1	ubqt	ubqt	ubqt	ubqt, (iYSL)	2.044	0.048	AF2F	no WISH report
cldnc	ubqt	ubqt	ubqt	ubqt, (iYSL)	1.614	0.045	AF2G	1-4 somite, Thisse, C., and Thisse, B. (2005) High Throughput Expression Analysis of ZF-Models Consortium Clones. ZFIN Direct Data Submission.
ef1	ubqt	ubqt	ubqt	ubqt	1.155	0.050	NS	1-4 somite, Thisse, C., and Thisse, B. (2005) High Throughput Expression Analysis of ZF-Models Consortium Clones. ZFIN Direct Data Submission.
grhl2a	ubqt	ubqt	ubqt, (iYSL)	ubqt,	1.604	0.040	AF2H	no WISH report
max	ubqt	ubqt	ubqt	ubqt	1.483	0.050	AF2H	1 cell, Thisse, B., Thisse, C. (2004) Fast Release Clones: A High Throughput Expression Analysis. ZFIN Direct Data Submission.
sart3	ubqt	ubqt	ubqt	ubqt	1.242	0.046	AF2K	1 cell, Thisse, B., Thisse, C. (2004) Fast Release Clones: A High Throughput Expression Analysis. ZFIN Direct Data Submission.
sema4e	ubqt	ubqt	ubqt, (iYSL)	ubqt	2.654	0.045	AF2E	no WISH report
wu:fc38c08	ubqt	ubqt	ubqt	ubqt	1.469	0.044	AF2I	no WISH report
wu:fc44h08	ubqt	ubqt	ubqt	ubqt	1.318	0.034	NS	no WISH report
zgc:113026	ubqt	ubqt	ubqt	ubqt	1.464	0.045	AF2I	1 cel, Thisse, B., Thisse, C. (2004) Fast Release Clones: A High Throughput Expression Analysis. ZFIN Direct Data Submission..
zgc:158225	ubqt	ubqt	ubqt	ubqt	1.839	0.047	AF2C	no WISH report
**No expression detected**								
ch25hl1.1	-	-	-	-	1.108	0.040	NS	gastrula and early somitogenesis, Thisse, B., Thisse, C. (2004) Fast Release Clones: A High Throughput Expression Analysis. ZFIN Direct Data Submission.
egr2a	(ubqt)	(ubqt)	(ubqt)	(ubqt)	1.684	0.042	AF2D	1-4 somite, Sun, Z., Shi, K., Su, Y., and Meng, A. (2002) A novel zinc finger transcription factor resembles krox-20 in structure and in expression pattern in zebrafish. Mech. Dev. 114(1-2): 133.
mpz	-	-	-	-	1.637	0.042	NS	50%, Thisse, B., Thisse, C. (2004) Fast Release Clones: A High Throughput Expression Analysis. ZFIN Direct Data Submission.
samd13	-	-	-	-	1.636	0.037	NS	no WISH report
six4.3	-	-	-	-	3.041	0.046	NS	gastrula, Kobayashi, M., Osanai, H., Kawakami, K., and Yamamoto, M. (2000) Expression of three zebrafish Six4 genes in the cranial sensory placodes and the developing somites. Mech. Dev. 98(1-2): 151-155.
wu:fb75e06	(deep)	-	-	-	1.252	0.044	NS	no WISH report
wu:fc35e07	-	-	-	-	1.241	0.044	NS	no WISH report
wu:fc45e06	-	-	-	-	1.644	0.042	NS	no WISH report
wu:fc96f03	-	-	-	-	1.374	0.042	NS	no WISH report
wu:fd60d11	-	-	-	-	1.490	0.045	NS	no WISH report
wu:fj65c07	-	-	-	-	4.855	0.046	NS	no WISH report
