# Supplementary material for: Pre-gastrula expression of zebrafish extraembryonic genes
Source: BMC Dev Biol. 2010 Apr 27;10:42. doi: 10.1186/1471-213X-10-42 (PMC2873407; doi:10.1186/1471-213X-10-42)

Additional File 2A

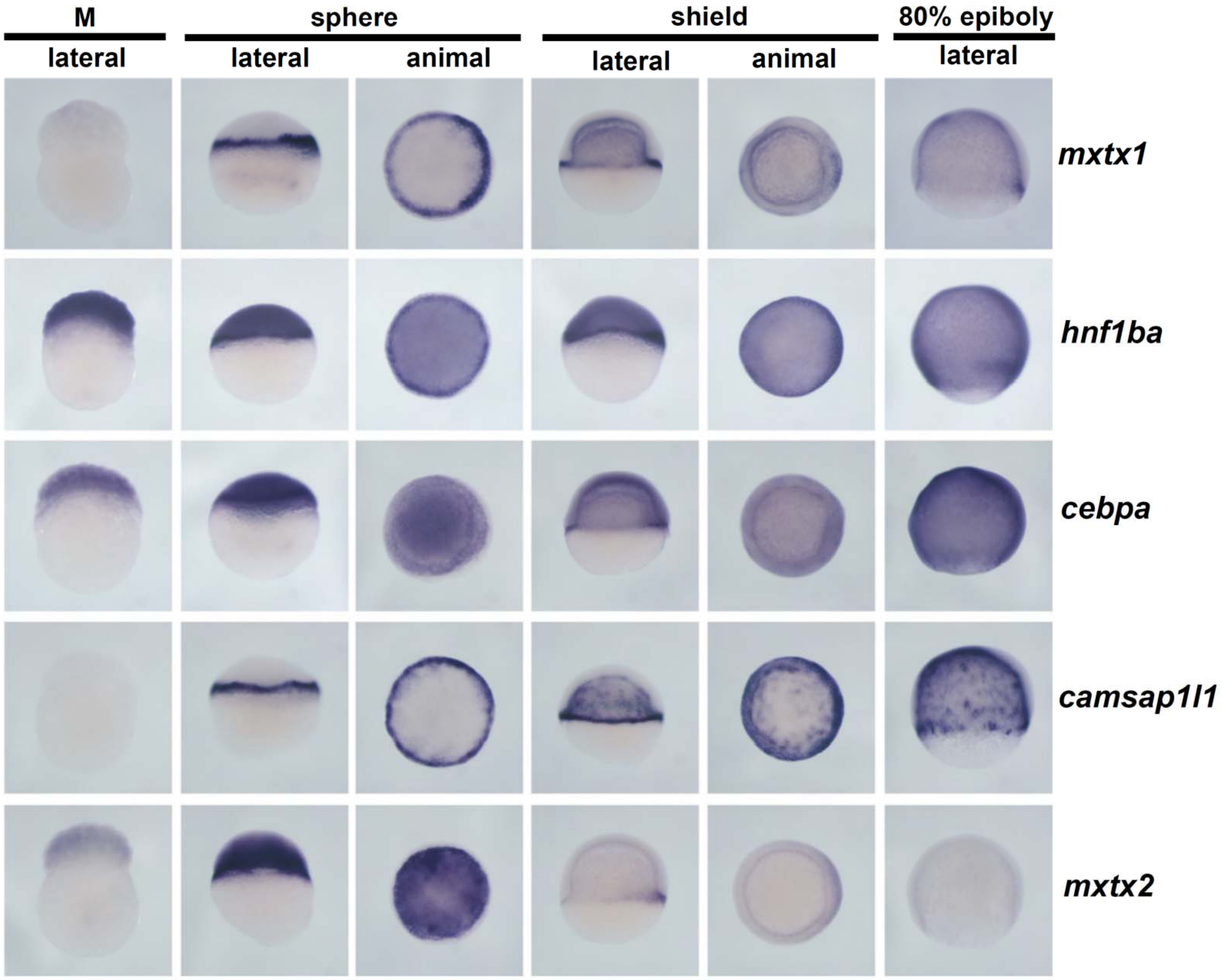

Additional File 2B

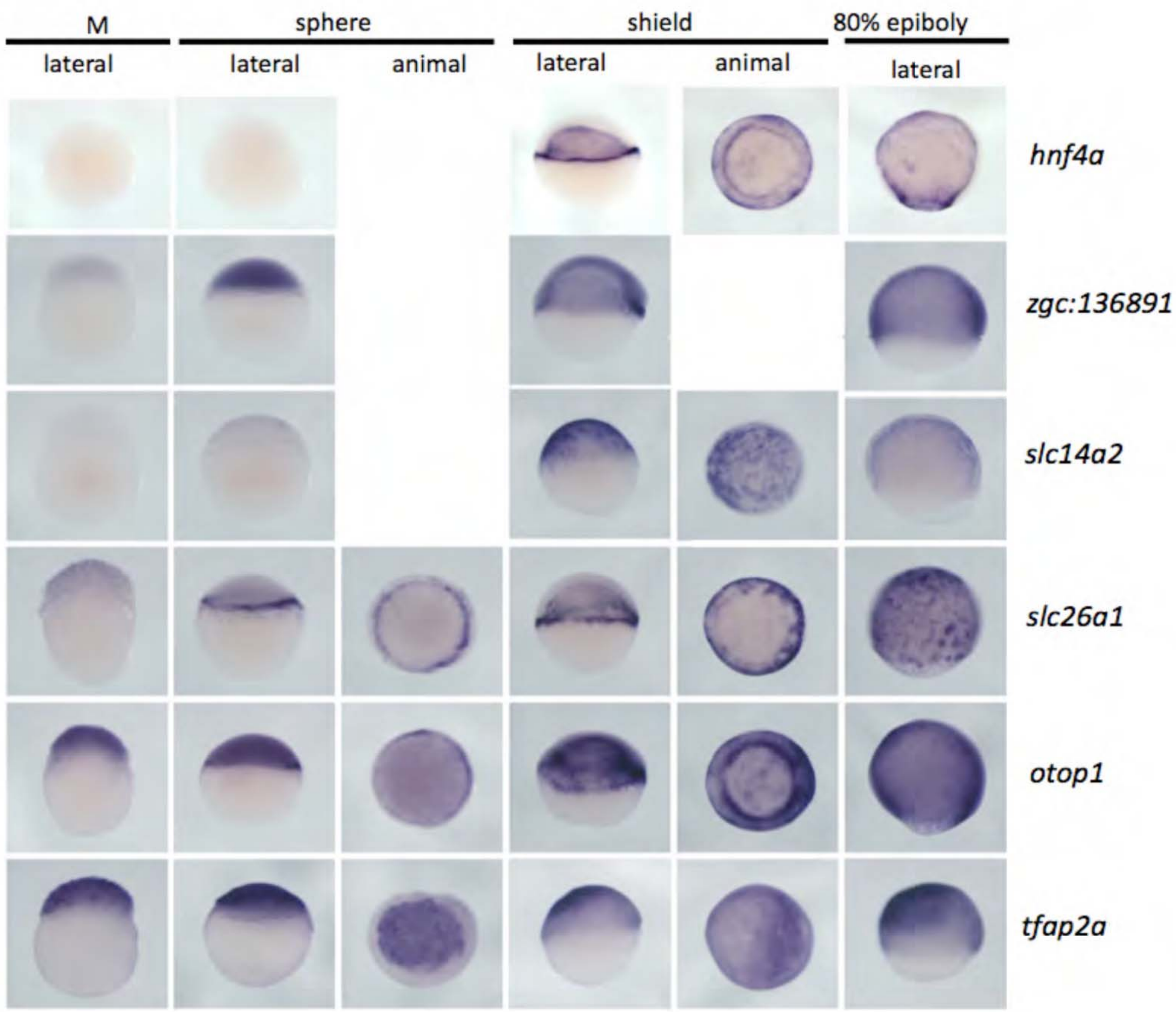

Additional File 2C

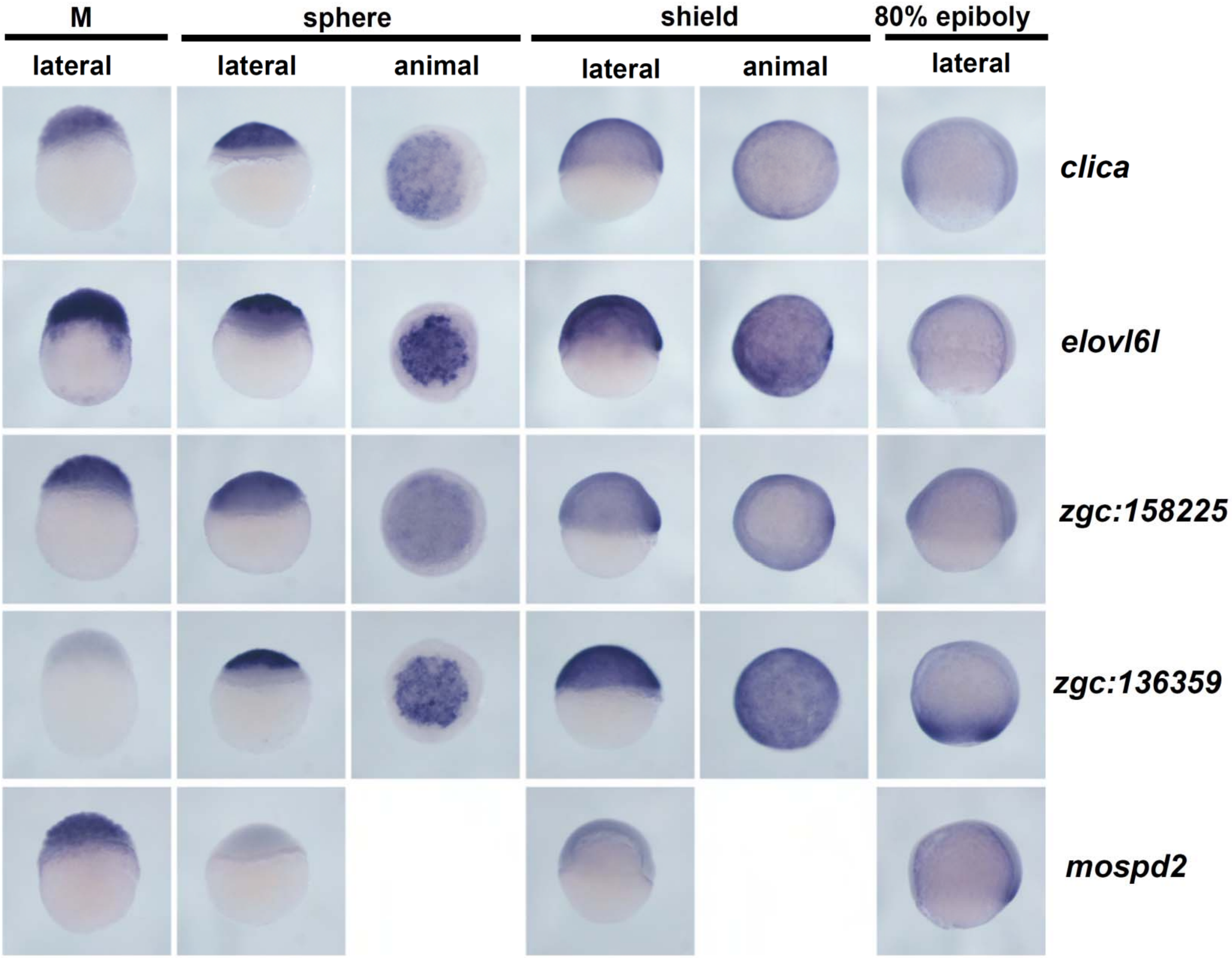

Additional File 2D

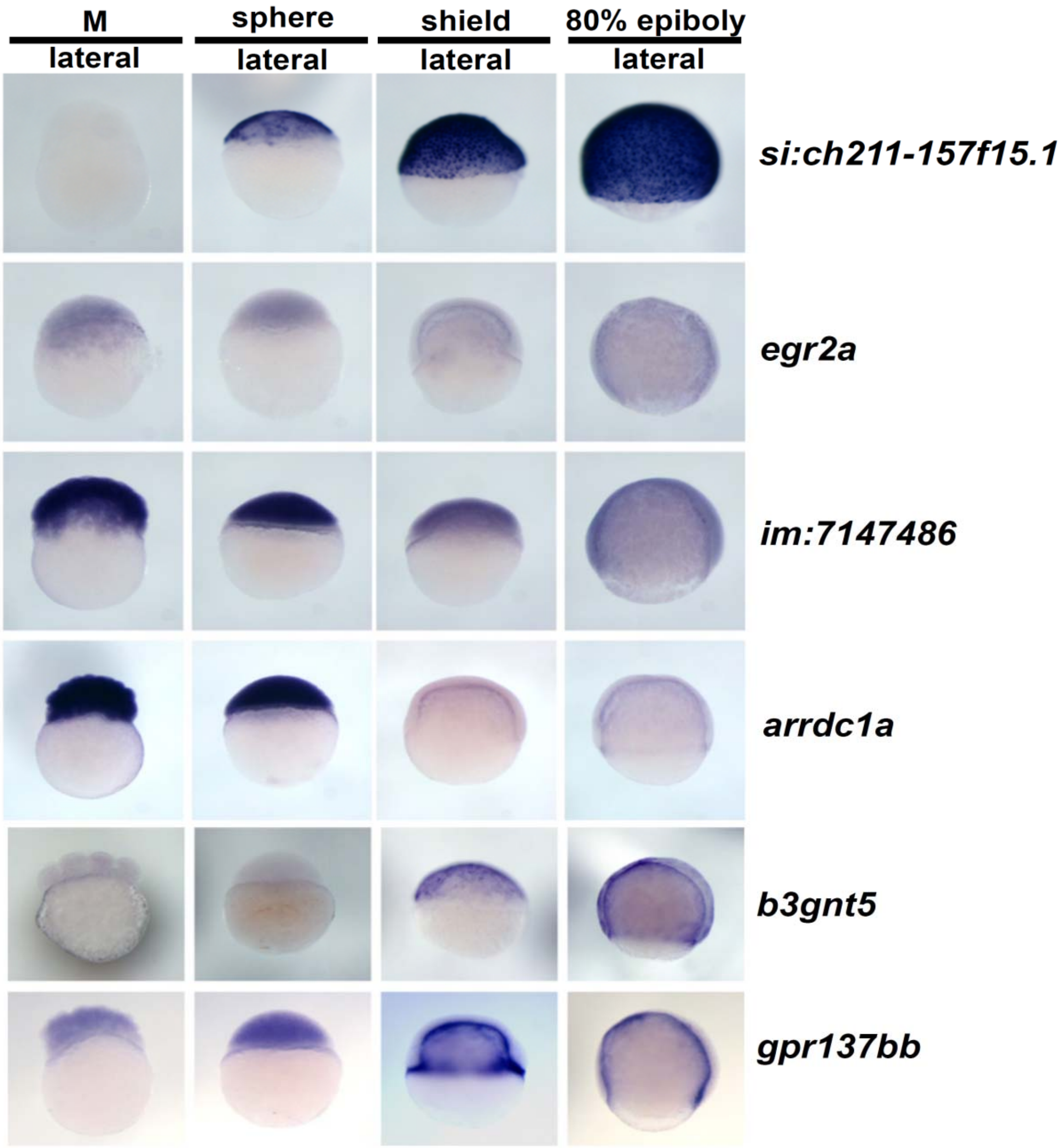

Additional File 2E

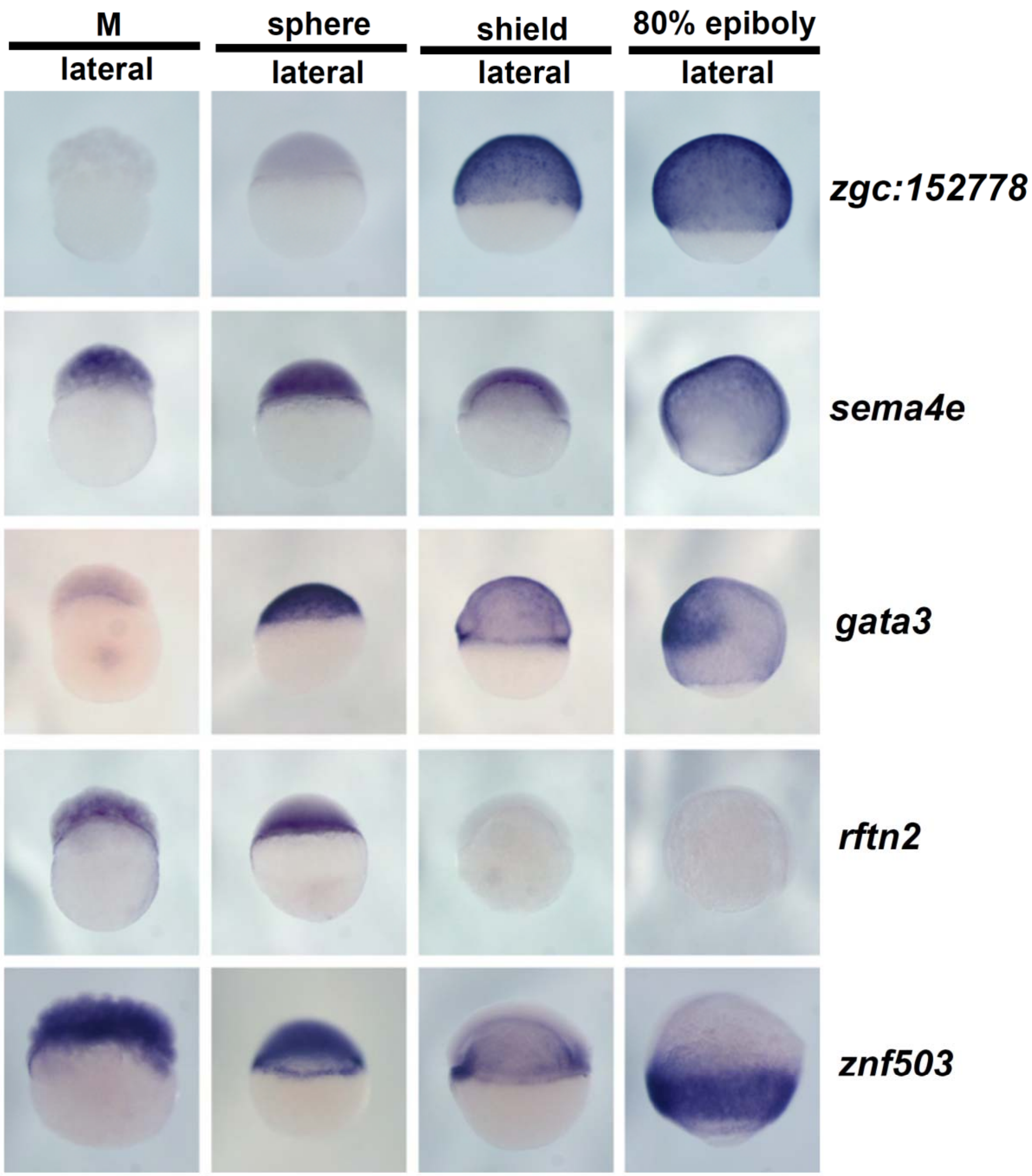

Additional File 2F

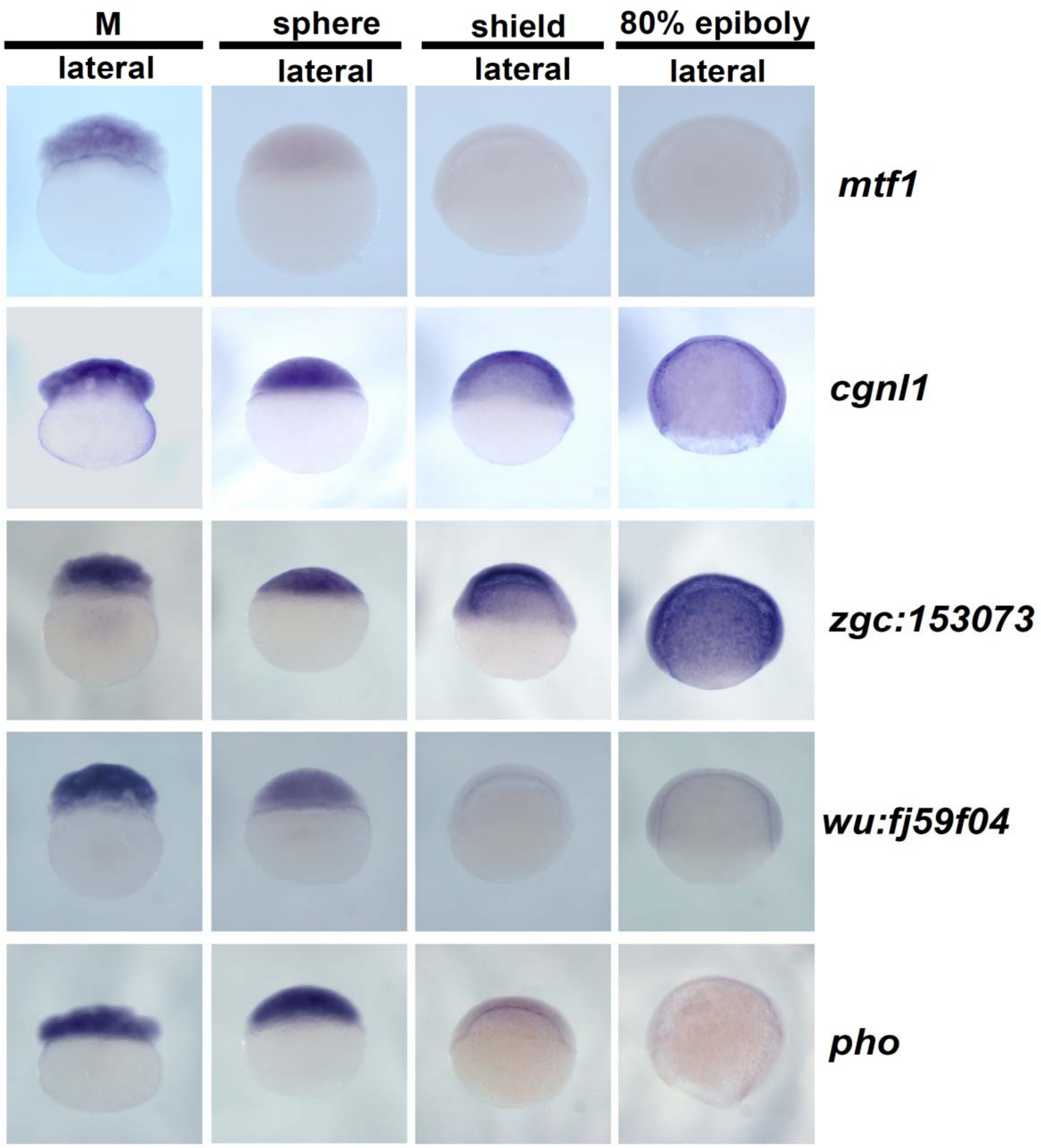

Additional File 2G

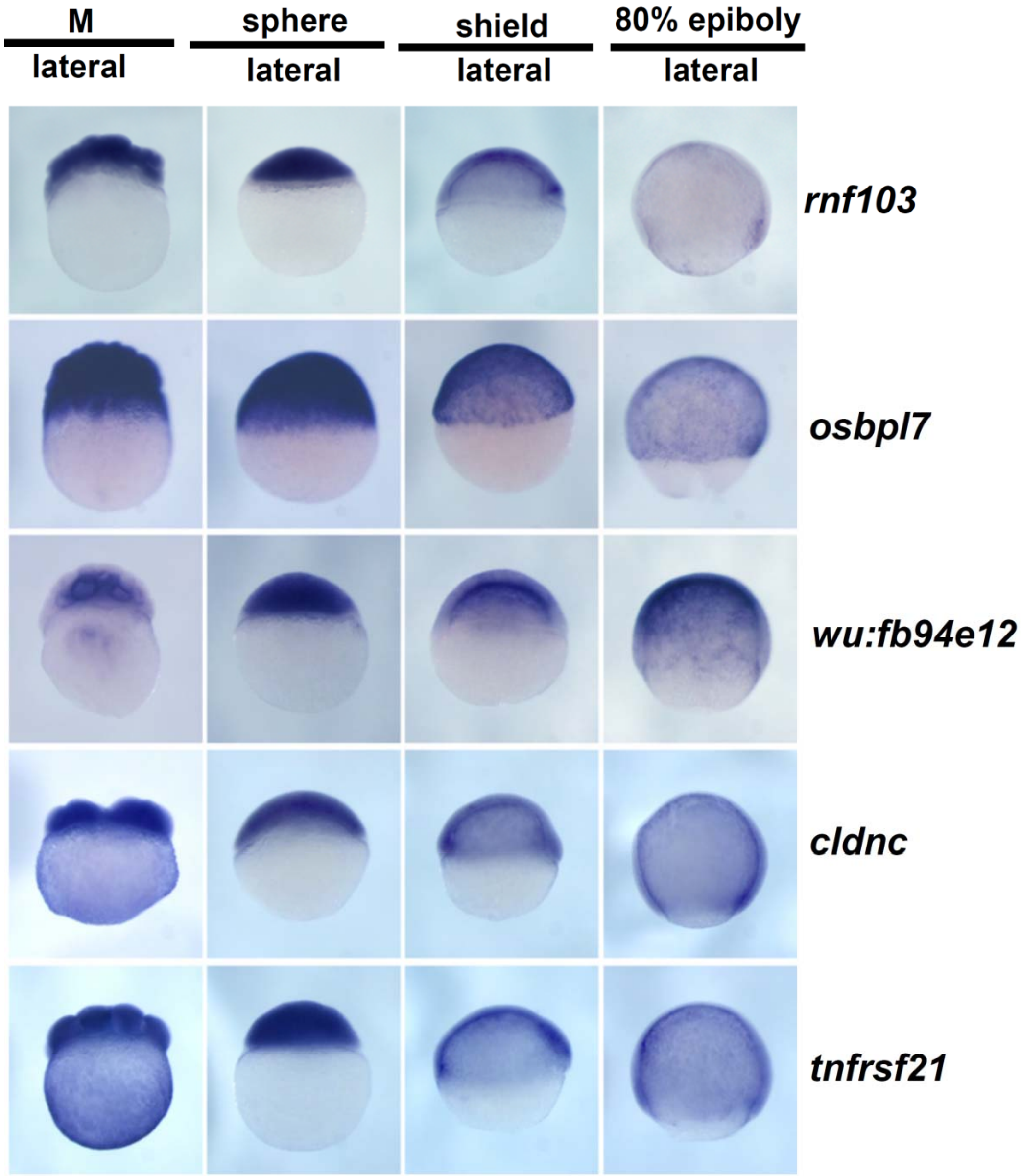

Additional File 2H

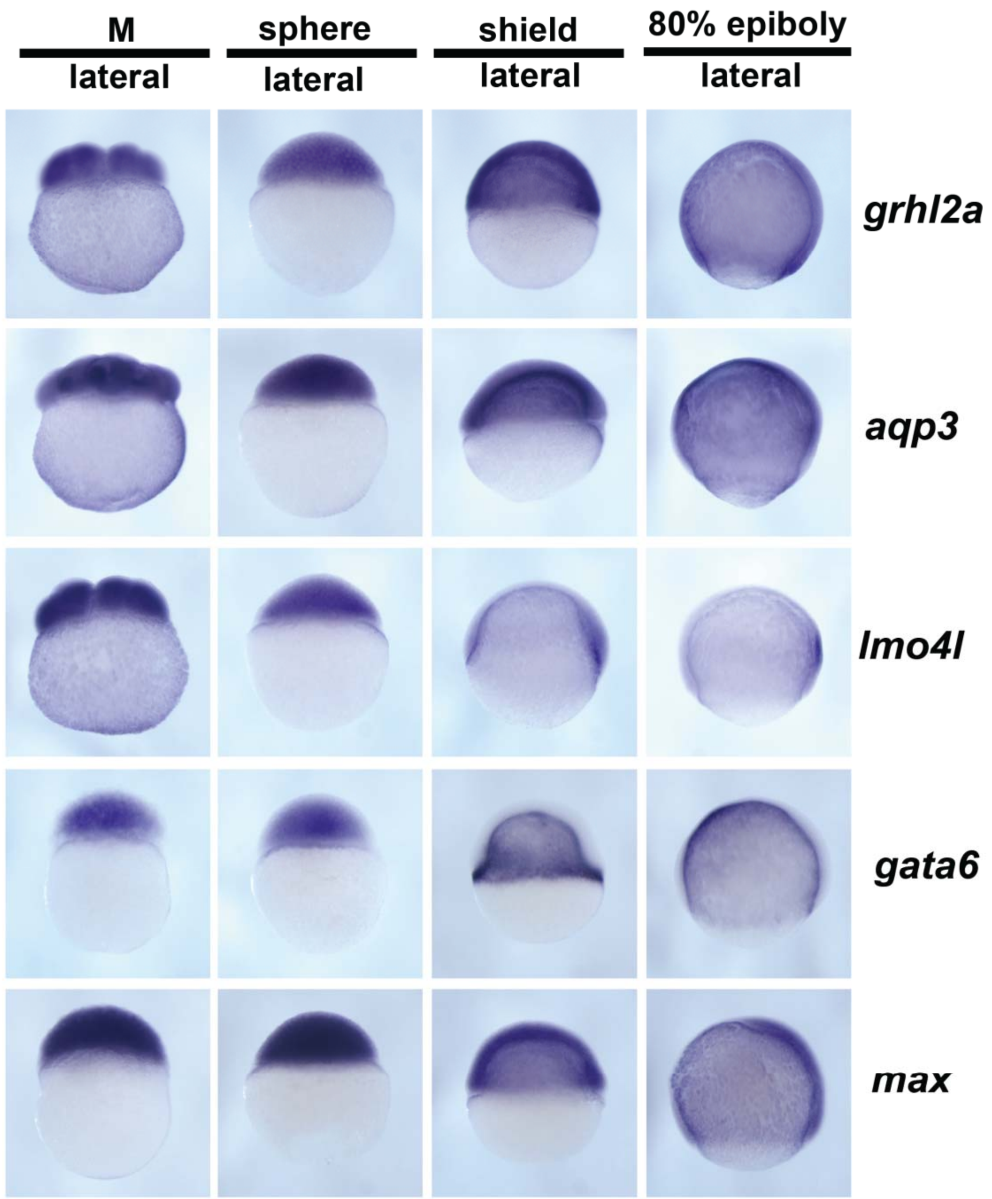

Additional File 2I

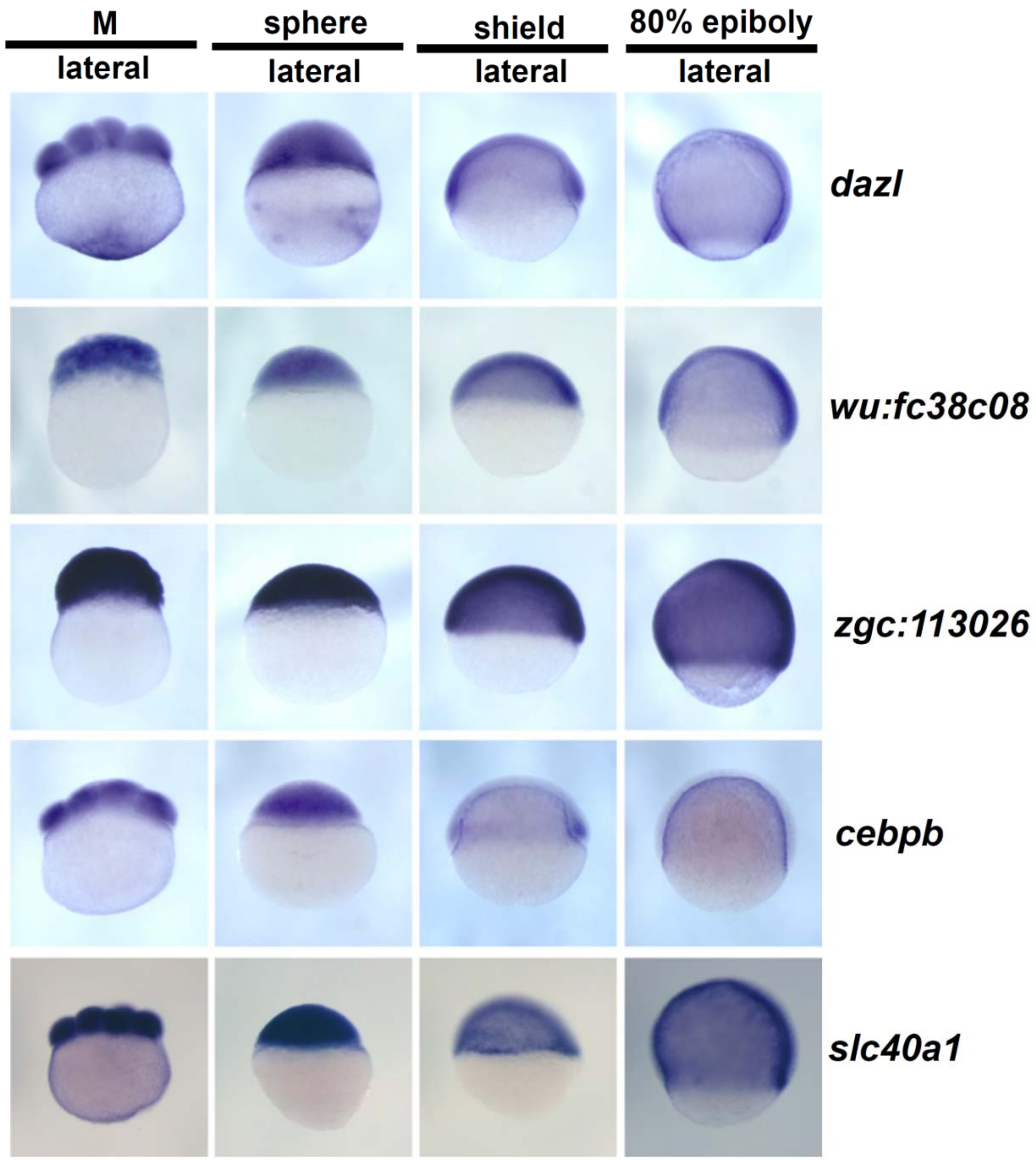

Additional File 2J

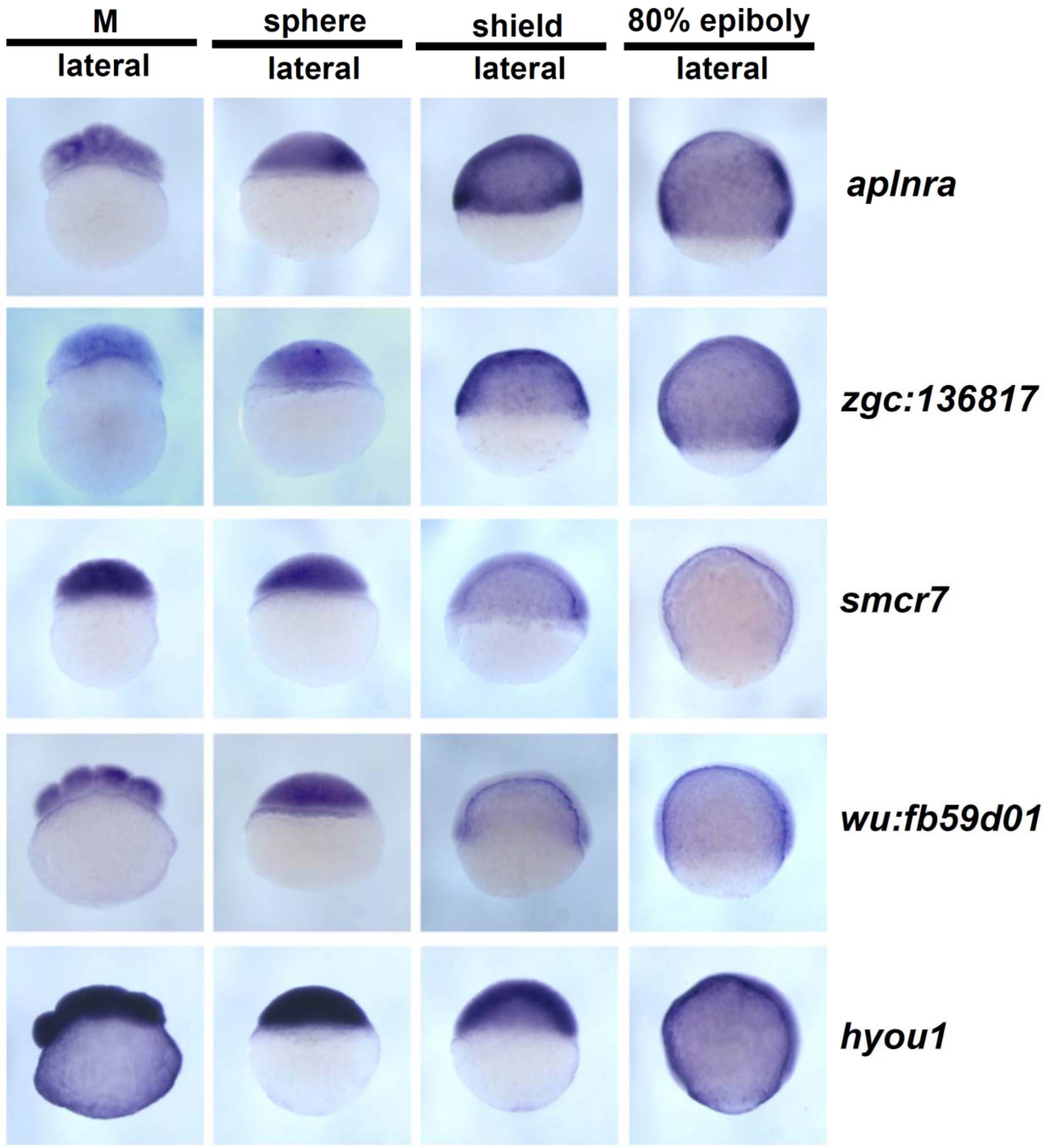

Additional File 2K

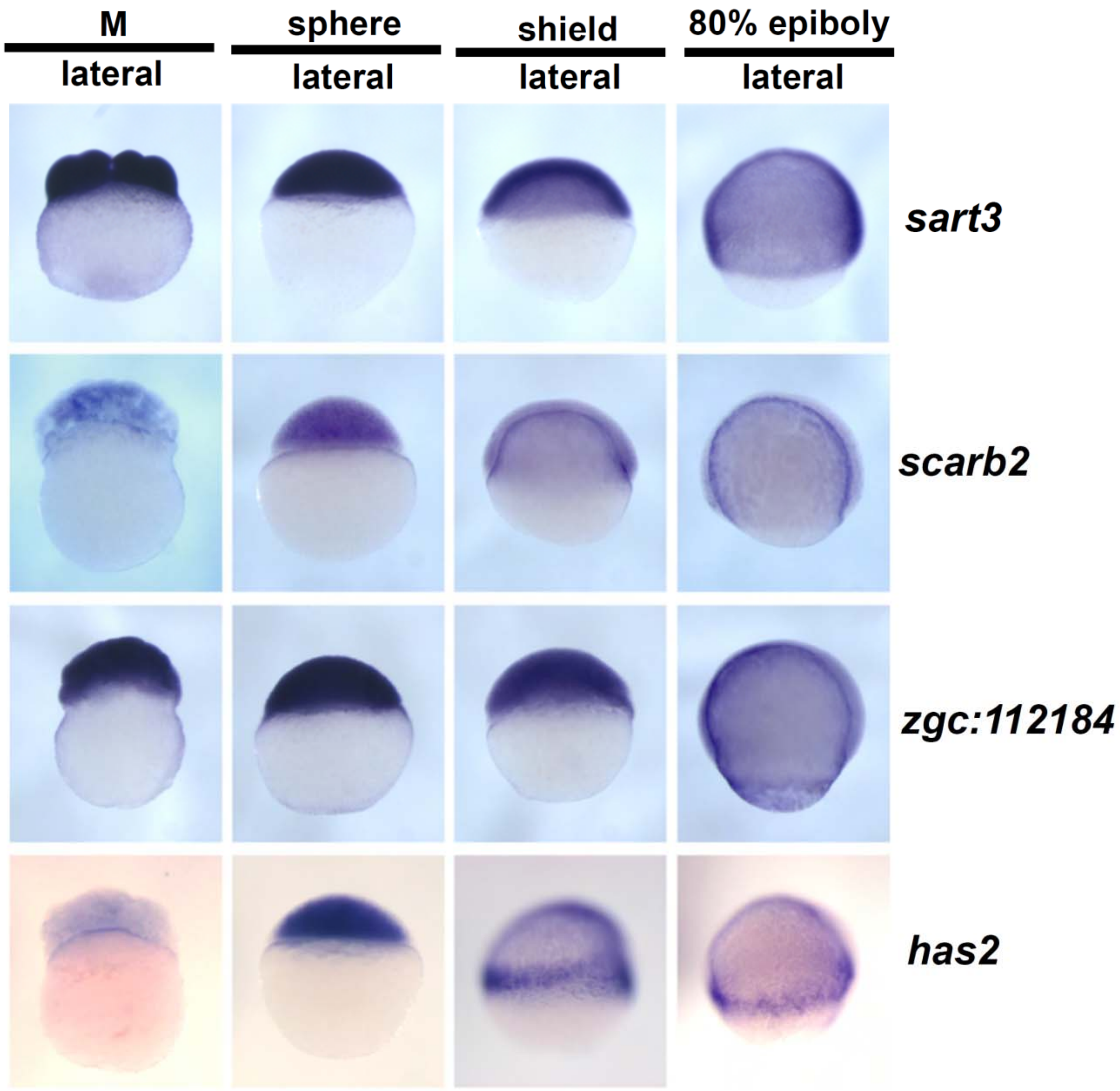

Supplement: Additional file 2 — Whole-mount in situ hybridization images for 56 genes. Two hundred and forty images representing four stages with some multiple views for each of fifty-six genes are presented on 11 pages (A through K). The location of data for particular genes is indicated in Additional File 1. No data is shown for ten genes for which no expression was detected and two genes that showed ubiquitous expression. [file 1471-213X-10-42-S2.PDF]
